# Age, Gender and Geographic Differences in Global Health Burden of Cirrhosis and Liver Cancer due to Nonalcoholic Steatohepatitis

**DOI:** 10.7150/jca.52282

**Published:** 2021-03-15

**Authors:** Tianyue Zhang, Jingya Xu, Lingxia Ye, Xiling Lin, Yufeng Xu, Xiaowen Pan, Xifang Weng, Chuyu Ye, Longjiang Fan, Yuezhong Ren, Peng-Fei Shan

**Affiliations:** 1 Department of Endocrinology, the Second Affiliated Hospital of Zhejiang University School of Medicine, 88 Jiefang Road, Hangzhou, Zhejiang, 310009, China.; 2 Department of Ophthalmology, the Second Affiliated Hospital of Zhejiang University School of Medicine, 88 Jiefang Road, Hangzhou, Zhejiang, 310009, China.; 3 Institute of Crop Sciences and Institute of Bioinformatics, Zhejiang University, Hangzhou, Zhejiang, 310058, China.

**Keywords:** nonalcoholic steatohepatitis, cirrhosis, liver cancer, disability-adjusted life years, health burden, Sociodemographic Index

## Abstract

**Objective:** Recently, Nonalcoholic Steatohepatitis (NASH) has become a major contributor to cirrhosis and liver cancer. Therefore, the Global Burden of Disease (GBD) 2017 was used to comprehensively analyze the global, regional, and national burden of cirrhosis and liver cancer due to NASH between 1990 and 2017.

**Methods:** Data for cirrhosis and liver cancer due to NASH were extracted from the GBD study 2017. Socio-demographic Index (SDI) in 2017 was cited as indicators of socioeconomic status. ARIMA model was established to forecast the future health burden. Kruskal-Wallis test and Pearson linear correlation were adopted to evaluate the gender disparity and association with socioeconomic level.

**Results:** From 1990-2017, the global disability-adjusted life years (DALYs) numbers of liver cancer due to NASH increased from 0.71 million to 1.46 million. The age-standardized DALYs rates of liver cancer due to NASH were negatively associated with SDI levels (r=0.-409, p<0.001). Geographically, Australasia experienced the largest increase in the burden of liver cancer due to NASH, with the age-standardized DALYs rate increasing by 143.54%. The global prevalence number of liver cancer due to NASH peaked at 60-64 years in males and at 65-69 years in females. Globally, the burden was heavier in males compared with females. Male-female-ratio of age-standardized DALYs rates in liver cancer due to NASH were positively related to SDI (r=0.303, P=0.011).

**Conclusion:** The global burden of NASH-associated liver cancer has increased significantly since 1990, with age, gender and geographic disparity. Public awareness of liver diseases due to NASH should be emphasized.

## Introduction

Nonalcoholic fatty liver disease (NAFLD) is a group of acquired metabolic-related liver diseases characterized by the presence of hepatic fat accumulation and the exclusion of other causes of hepatic steatosis 1. With the rising prevalence of obesity, NAFLD has become a heavy burden for personal and public health 1, 2. Nonalcoholic steatohepatitis (NASH) occurs in approximately 59% of patients with NAFLD, which is diagnosed by liver biopsy findings of chronic liver injury and inflammation plus steatosis 3. NASH is rapidly becoming a major contributor to cirrhosis and liver cancer 4-6. In an 8-year period, 21-26% of NASH progressed to cirrhosis and 13% progressed directly to liver cancer 7-9.

To our knowledge, few studies on the burden of cirrhosis and liver cancer due to NASH by time, age, gender, region or socioeconomic status have been published. The health burden of cirrhosis and liver cancer due to NASH has been quantified by disability-adjusted life years (DALYs) in the Global Burden of Disease (GBD) study 10. DALYs, defined as the sum of years lived with disability and years of life lost owing to premature death, combined both the prevalence of a disease and its impact on mortality and morbidity 10. Thus, it could illustrate the whole picture of a disease and provide hints for public health policy development. Accordingly, in the present study, we sought to investigate the changes in global health burden of cirrhosis and liver cancer due to NASH from 1990 to 2017, and the age, gender and geographic differences in the health burden, based on the GBD study 2017.

## Materials and Methods

### Data source

The GBD study 2017 provides a detailed epidemiologic assessment (including incidence, prevalence, mortality and DALYs etc.) of 345 diseases and injuries and 84 risk factors by age and sex on a global scale and for 195 countries and territories spanning the years from 1990 to 2017. The detailed methods about inputs, analytical processes, and outputs have been described in previous paper elsewhere 11-16. Briefly, the study used a total of 68,781 data sources (including published literature, surveillance data, survey data, hospital and clinical data, and other types of data), maximized the comparability of data and provided a standard approach for estimating disease burden. Citations for all data sources in GBD 2017 are provided in searchable form through a web tool (https://gbd2017.healthdata.org/gbd-search/). An integrative Bayesian meta-regression method that estimates a generalized negative binomial model for all epidemiological data was used through DisMod-MR 2.1 in the computation of GBD estimates of disease burden.

Data for cirrhosis and liver cancer due to NASH were extracted from the GBD study 2017. In GBD study 2017, cirrhosis and liver cancer due to NASH were defined only if NASH was listed as a specific etiology for cirrhosis and liver cancer. All GBD data used in this study were collected from the Global Health Data Exchange 10 which comprised: (1) Global total and gender-specific data on incidence, prevalence, death and DALYs, as absolute numbers, and age-standardized rates (per 100 000 population), annually from 1990 to 2017; (2) Global gender-age-specific prevalence and DALYs data, as absolute numbers in 2017; (3) GBD regions' total and gender-specific DALYs data in 1990 and 2017, as age-standardized rates; (4) National total and gender-specific DALYs data, as absolute numbers and age-standardized rates in 2017; (5) Gender-specific age-standardized DALYs rates by Socio-demographic Index (SDI) levels in 2017. Ethical approval and informed consent were not required for this study because of public accessibility of the data.

### Socioeconomic status

The SDI was calculated by the GBD study 2017 as a composite indicator of development status strongly correlated with health outcomes 10. In short, it is the geometric mean of 0 to 1 indices of total fertility rate under the age of 25 (TFU25), mean education for those aged 15 and older (EDU15+), and lag distributed income (LDI) per capita, with a higher value implying a higher level of socioeconomic development. Countries or territories were divided into five groups based on their SDI values in 2017 (See the Supplementary Table S3): High SDI (> 0.81), high-middle SDI (0.70-0.81), middle SDI (0.61-0.70), low-middle SDI (0.46-0.60), and low SDI (< 0.46).

### Forecasting cirrhosis and liver cancer due to NASH burden beyond 2017

Auto-Regressive Integrated Moving Average (ARIMA) model was widely used in epidemiological study for forecasting future trend 17. ARIMA model was established in R language to forecast cirrhosis and liver cancer due to NASH burden from 2018 towards 2050 (See the Supplementary Statistical Analysis Methods for details).

### Statistical analysis

Data are expressed as a value with a 95% uncertainty interval (UI). Age-standardized rates are expressed as the number per 100 000 population. The Kruskal-Wallis test was adopted to assess differences in global age-standardized prevalence and DALYs rates for each SDI-based country group. Association of gender difference (male-female ratio) in age-standardized DALYs rates with SDI were tested via Pearson correlation and Linear regression analyses. All statistical analyses, except as otherwise specified, were conducted with Prism software (version 8; GraphPad). A P-value less than 0.05 was considered statistically significant.

## Results

### Temporal trends of burden of cirrhosis and liver cancer due to NASH from 1990 to 2017

**Figure 1:**
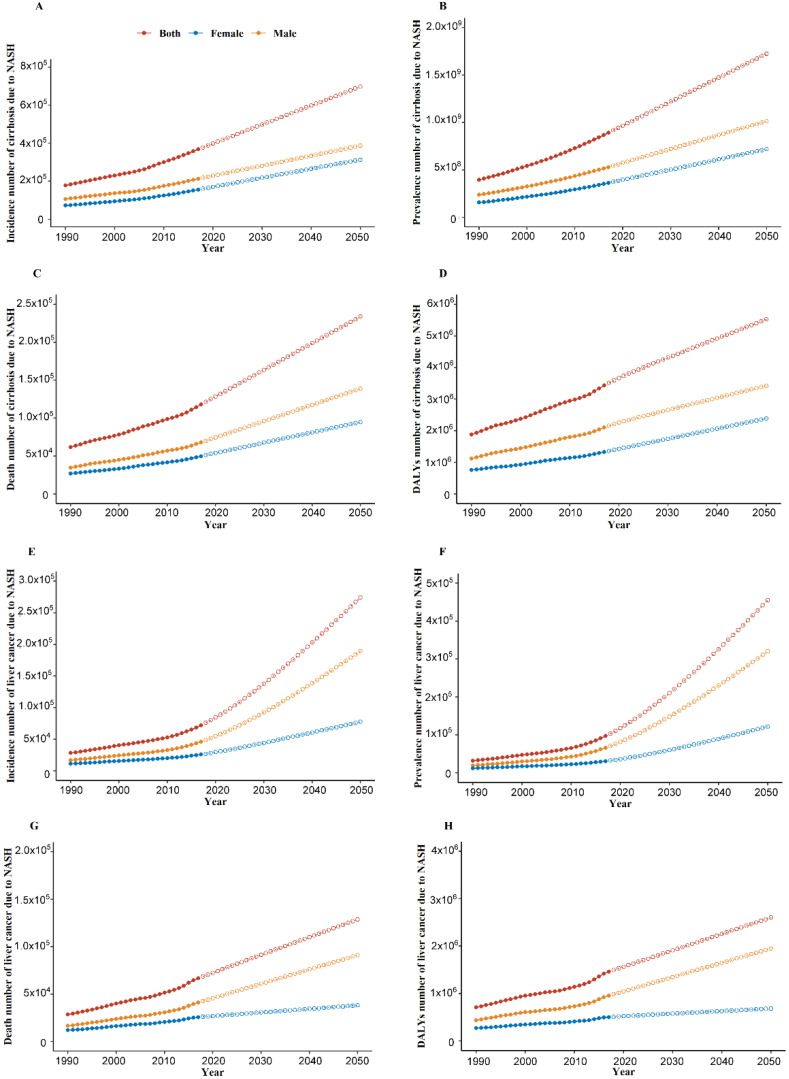
Global burden of cirrhosis and liver cancer due to NASH from 1990 to 2050. (A) Incidence number of cirrhosis due to NASH; (B) Prevalence number of cirrhosis due to NASH; (C) Death number of cirrhosis due to NASH; (D) DALYs number of cirrhosis due to NASH; (E) Incidence number of liver cancer due to NASH; (F) Prevalence number of liver cancer due to NASH; (G) Death number of liver cancer due to NASH; (H) DALYs number of liver cancer due to NASH. NASH: nonalcoholic steatohepatitis; DALYs: disability-adjusted life years. Dash line: forecasted NASH burden from 2018 towards 2050 through ARIMA model.

As shown in Figure 1 and Figure S1, from 1990-2017, increasing trends of global burden of cirrhosis and liver cancer due to NASH were observed in incidence number, prevalence number, death number, DALYs number, age-standardized incidence rate and age-standardized prevalence rate. For the global age-standardized death rate and DALYs rate of cirrhosis due to NASH, we observed a decrease from 2005 to 2013 and an increase from 2013 to 2017. Meanwhile, for the global age-standardized death rate and DALYs rate of liver cancer due to NASH, we observed a decrease from 2003 for death rate and 2000 for DALYs rate to 2007, a plateau from 2007 to 2013, and an increase from 2013 to 2017.

Overall, as shown in Table 1, the global all-age incidence of cirrhosis due to NASH was 367,779 (334,460-403,729) cases with an age-standardized rate of 4.6 (4.2-5.1) per 100,000 in 2017. The cirrhosis due to NASH prevalence cases were 892.32 million (95% UI: 858.62-927.95 million) and the age-standardized rate was 11,060.5 (95% UI: 10651.3-11493.1) per 100,000 in 2017. The cirrhosis due to NASH death number was 118,030 (108,618-128,577) with an age-standardized rate of 1.5 (1.3-1.6) per 100,000 in 2017. As for DALYs, the all-age DALYs numbers were 3.43 million (95% UI: 3.15-3.74 million) with an age-standardized rate of 41.8 (38.5-45.5) per 100,000 in 2017.

The global all-age incidence of liver cancer due to NASH was 72,197 (64,602-79,915) cases with an age-standardized rate of 0.9 (0.8-1.0) per 100,000 in 2017. The liver cancer due to NASH prevalence cases were 97,376 (95% UI:86,789-108,049) and the age-standardized rate was 1.2 (1.1-1.3) per 100,000 in 2017. The liver cancer due to NASH death number was 66,875 (59,560-74,511) with an age-standardized rate of 0.8 (0.8-0.9) per 100,000 in 2017. As for DALYs, the all-age DALYs numbers were 1.46 million (95% UI: 1.30-1.62 million) with an age-standardized rate of 18.0 (16.1-20.0) per 100,000 in 2017.

### Age and gender differences in burden of cirrhosis and liver cancer due to NASH

**Figure 2:**
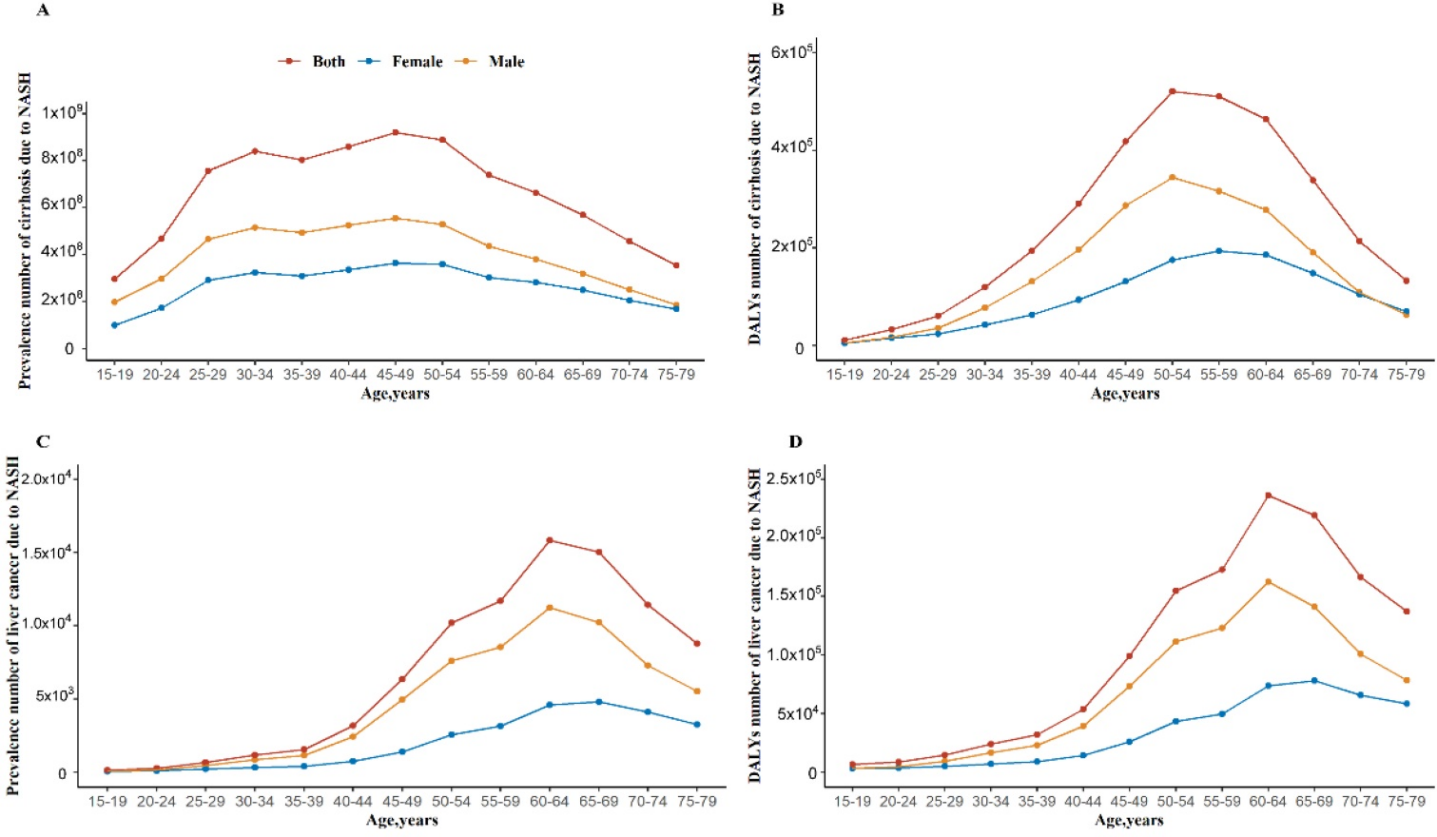
Global burden of cirrhosis and liver cancer due to NASH by age in 2017. (A) Prevalence number of cirrhosis due to NASH; (B) DALYs number of cirrhosis due to NASH; (C) Prevalence number of liver cancer due to NASH; (B) DALYs number of liver cancer due to NASH. NASH: nonalcoholic steatohepatitis; DALYs: disability-adjusted life years.

The age and sex-specific prevalence and DALYs number for cirrhosis and liver cancer due to NASH were shown in Figure 2. For both males and females, age group (45-49 years) had the highest prevalence numbers of cirrhosis due to NASH. For males, age group (60-64 years) had the highest prevalence numbers of liver cancer due to NASH. For females, age group (65-69 years) had the highest prevalence numbers of liver cancer due to NASH. The prevalence and DALYs number were higher in males than females. At older age groups above 70 years, the DALYs number in females for cirrhosis due to NASH started to be higher than males.

### Geographic differences in burden of cirrhosis and liver cancer due to NASH

**Figure 3:**
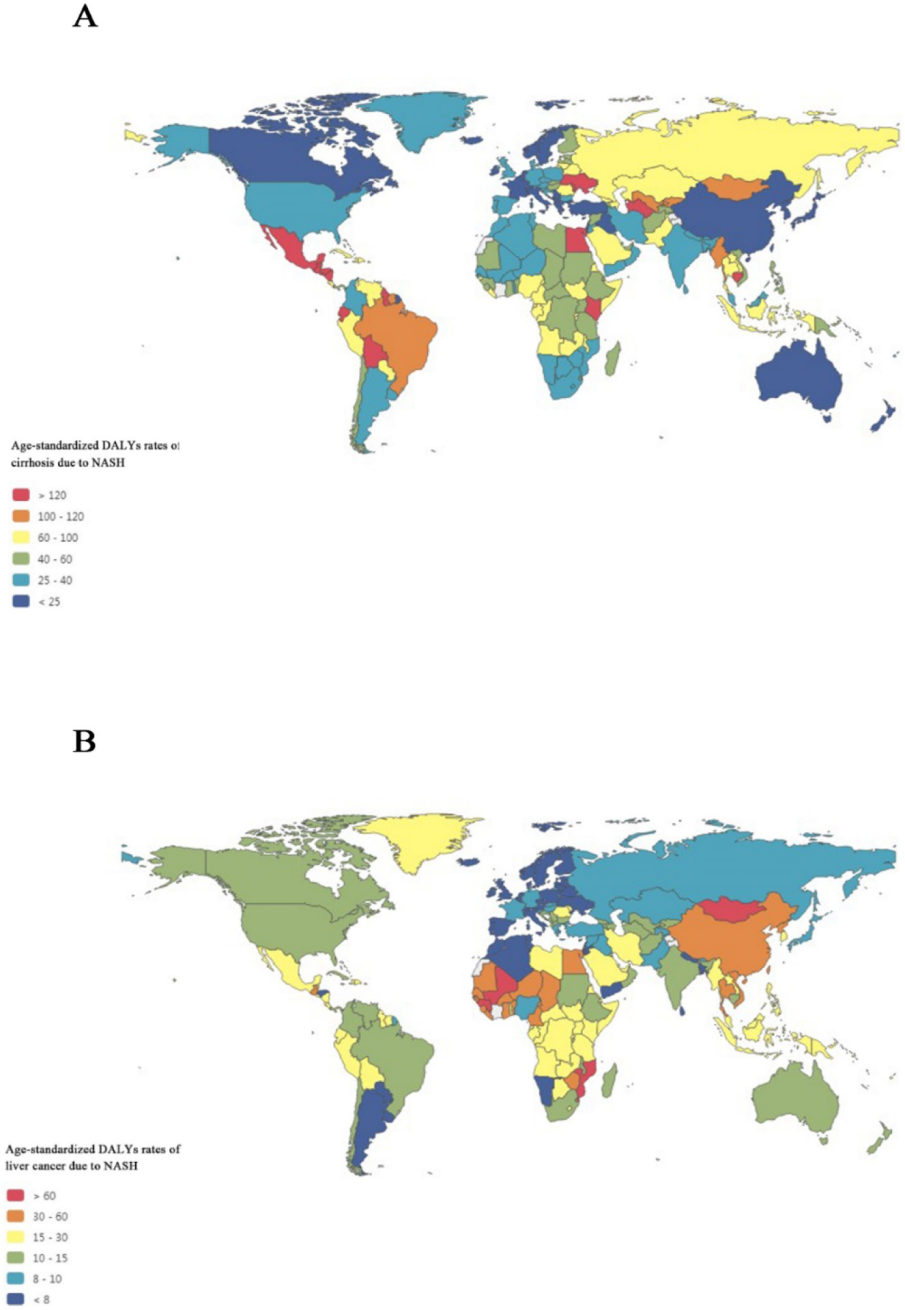
Global map of health burden of cirrhosis and liver cancer due to NASH in 2017. (A) Age-standardized DALYs rate of cirrhosis due to NASH; (B) Age-standardized DALYs rate of liver cancer due to NASH. NASH: nonalcoholic steatohepatitis; DALYs: disability-adjusted life years.

Figure 3A maps the distribution of health burden of cirrhosis due to NASH in 2017.The top five countries for the age-standardized DALYs rates of cirrhosis due to NASH were Egypt (239.9), Cambodia (203.2), Moldova (192.5), Guatemala (182.0), and Honduras (173.3), while the bottom five countries for the DALYs rates of cirrhosis due to NASH were Singapore (3.1), Japan (9.5), Brunei (11.3), Norway (11.9), and New Zealand (12.8).

Figure 3B maps the distribution of health burden of liver cancer due to NASH in 2017. Overall, the global distribution of health burden of cirrhosis due to NASH was different from that of liver cancer due to NASH. The top five countries for age-standardized DALYs rates of liver cancer due to NASH were Mongolia (148.2), Gambia (93.9), Mali (83.3), Guinea (81.6), and Tonga (76.5), while the bottom five countries for the DALYs rates of liver cancer due to NASH were Tunisia (3.4), Norway (3.4), Andorra (3.9), Malta (4.3), and Morocco (4.4).

### Burden of cirrhosis and liver cancer due to NASH by GBD regions

**Figure 4:**
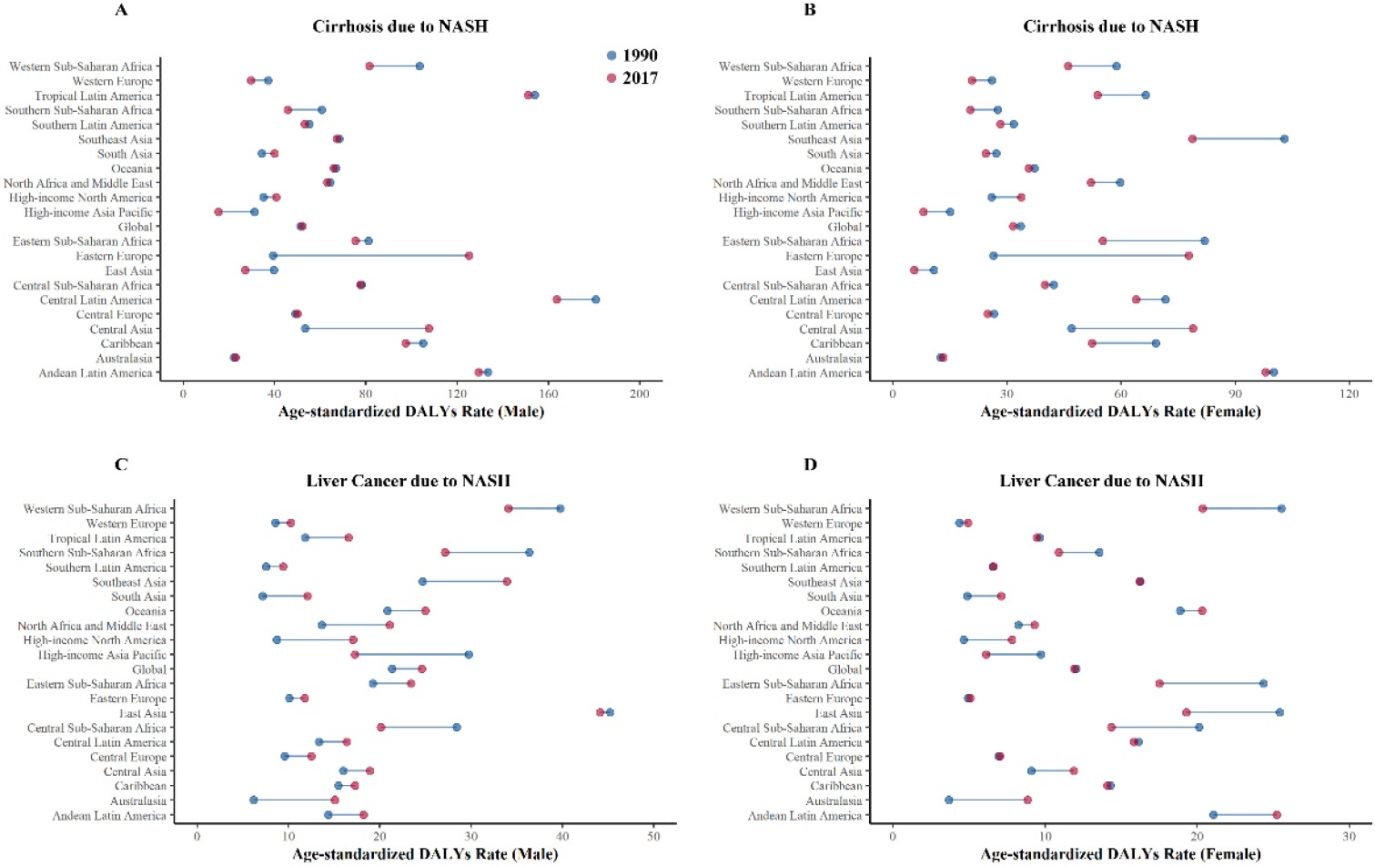
Health burden of cirrhosis and liver cancer due to NASH by GBD regions in 1990 and 2017. (A) Age-standardized DALYs rates of cirrhosis due to NASH (male); (B) Age-standardized DALYs rates of cirrhosis due to NASH (female); (C) Age-standardized DALYs rates of liver cancer due to NASH (male); (D) Age-standardized DALYs rates of liver cancer due to NASH (female). NASH: nonalcoholic steatohepatitis; DALYs: disability-adjusted life years.

We further looked at the health burden in each GBD regions in 2017 and compared with that in 1990 (Figure 4). Overall, Eastern Europe experienced the largest increase in the health burden of cirrhosis due to NASH, with the age-standardized DALYs rate increasing by 212.17%, while High-income Asia Pacific experienced the largest decrease in the health burden of cirrhosis due to NASH, with the age‑standardized DALY rats decreasing by 48.80%.

Australasia experienced the largest increase in the health burden of liver cancer due to NASH, with the age-standardized DALYs rate increasing by 143.54%, while High-income Asia Pacific experienced largest decrease in the health burden of liver cancer due to NASH, with the age-standardized DALYs rate decreasing by 39.77%. Detailed data of DALYs rates of cirrhosis and liver cancer due to NASH were displayed in Table 2.

### Burden of cirrhosis and liver cancer due to NASH by socioeconomic status

**Figure 5:**
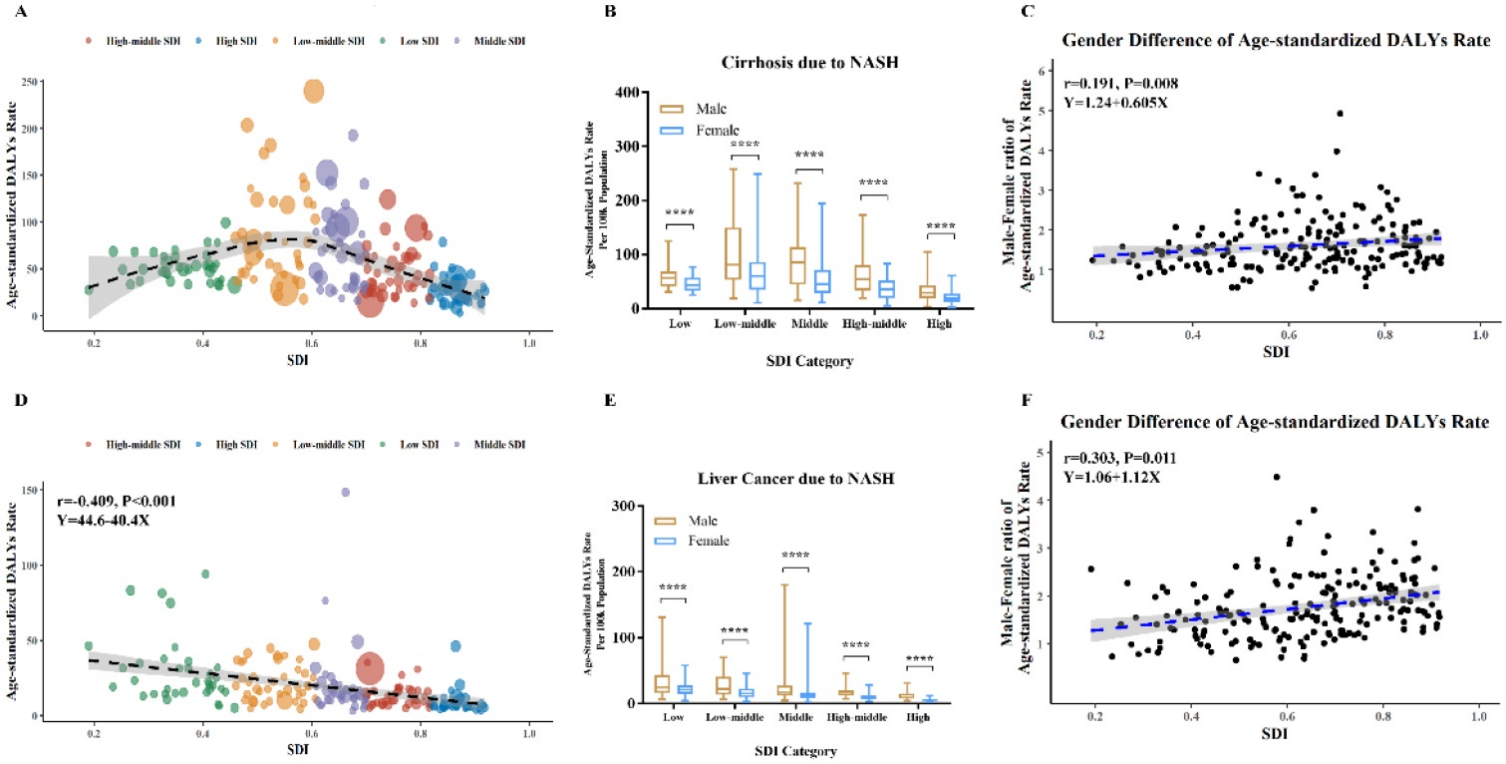
Global burden of cirrhosis and liver cancer due to NASH by SDI levels in 2017. (A) Age-standardized DALYs rate of cirrhosis due to NASH in 195 countries; (B) Age-standardized DALYs rate of cirrhosis due to NASH in different SDI levels; (C) Association between male-female-ratio of age-standardized DALYs rate of cirrhosis due to NASH with SDI; (D) Age-standardized DALYs rate of liver cancer due to NASH in 195 countries; (E) Age-standardized DALYs rate of liver cancer due to NASH in different SDI levels; (F) Association between male-female-ratio of age-standardized DALYs rate of liver cancer due to NASH with SDI. NASH: nonalcoholic steatohepatitis; SDI: sociodemographic index; DALYs: disability-adjusted life years. (A and D) Each circle represents a country; circles are colored according to SDI quintile; circle size corresponds to the number.

Bubbles in Figure 5A exhibited health burden of cirrhosis due to NASH in each country or territory with different SDI levels. The area size of each bubble represented DALYs number and Y coordinate indicated age-standardized DALYs rate. The highest DALYs number of cirrhosis due to NASH occurred in India (355,010). Meanwhile, the highest age-standardized rates of cirrhosis due to NASH occurred in Egypt (239.9). The age-standardized rates of cirrhosis due to NASH showed an inverted U-shaped relation with SDI levels, with low-middle SDI countries having the heaviest burden.

Bubbles in Figure 5D exhibited health burden of liver cancer due to NASH in each country or territory with different SDI levels. The highest DALYs number of liver cancer due to NASH occurred in China (621,923). Meanwhile, the highest age-standardized rates of liver cancer due to NASH occurred in Mongolia (148.2). The age-standardized rates of liver cancer due to NASH was negatively associated with SDI levels in Pearson correlation (r=-0.409, p<0.001) and in Linear regression (equation: Y=44.6-40.4*X).

As shown in Figure 5B, Figure 5E, male had higher age-standardized DALYs rates of cirrhosis and liver cancer due to NASH across all SDI categories. Differences by sex in age-standardized DALYs rates of cirrhosis due to NASH were positively associated with SDI in Pearson correlation (r=0.191, p=0.008) and in Linear regression (equation: Y=1.24+0.605*X) (Figure 5C). Besides, differences by sex in age-standardized DALYs rates of liver cancer due to NASH were positively associated with SDI as well in Pearson correlation (r=0.303, p=0.011) and in Linear regression (equation: Y=1.06+1.12*X) (Figure 5F).

## Discussion

The present study provides an overview of the global pattern of the health burden of cirrhosis and liver cancer due to NASH by year, gender, age, region, and socioeconomic levels. We found that the incidence number, prevalence number, death number, DALYs number, age-standardized incidence rate and age-standardized prevalence rate were increasing over time. The age-standardized rates of cirrhosis due to NASH showed an inverted U-shaped relation with SDI levels, with low-middle SDI countries having the heaviest burden. The age-standardized rates of liver cancer due to NASH was negatively associatedwith SDI levels. Gender disparity has existed since 1990, with male being more heavily impacted. Male-female-ratio of age-standardized DALYs rates in liver cancer due to NASH were positively related to SDI.

From 1999 to 2017, the global age-standardized incidence and prevalence rates of cirrhosis and liver cancer due to NASH increased dramatically, and measurements would keep rising if without further effective interventions, paralleling the dramatic modifications in lifestyle and the worldwide increase in obesity 18. Recent age-adjusted estimates of global obesity prevalence reported that at least 30% of male and 35% of female are obese in many countries worldwide 19. Conversely, the global age-standardized death and DALYs rates of cirrhosis and liver cancer due to NASH were not increasing at the same rate, which might be associated with recent advances in early diagnosis and therapy of cirrhosis and liver cancer 20, 21. It is worth noting, however, that there was an upward trend in the global age-standardized death and DALYs rates of liver cancer due to NASH in males according to ARIMA projection model. Despite all that, the global absolute numbers of incidence, prevalence, death and DALYs increased continuously from 1990 to 2017, which was probably related to population growth and aging 22.

Our study observed socioeconomic disparity in the burden of cirrhosis and liver cancer due to NASH. The age-standardized rates of cirrhosis due to NASH showed an inverted U-shaped relation with SDI levels, with low-middle SDI countries having the heaviest burden. The age-standardized rates of liver cancer due to NASH was negatively associated with SDI levels. One possible explanation might be that low SDI countries and low-middle SDI countries are undergoing a period of rapid economic and social changes, with trends towards urbanization and dramatic changes in diets and lifestyles, and these factors might increase the risks of obesity, NAFLD, and NASH, while people in high-SDI countries are more likely to have healthy diet habits 23, 24. What's more, with rapid urbanization in developing countries, air pollution caused by multiple-pollutant emissions and vehicle exhaust has been aggravated year by year. Compared with developed countries who were experienced in dealing with air pollution, developing countries may be less experienced. An increasing number of studies have shown that air pollution containing particulate matter (PM) ≤ 2.5 µm (PM2.5) plays an important role in the development of NAFLD 25. Evidence from a Taiwanese perspective cohort Study (REVEAL-HBV) further showed that long-term PM2.5 exposure increased the risk for liver cancer 26.

It should be noted that the global distribution of health burden of cirrhosis due to NASH was different from that of liver cancer due to NASH. For example, although the age-standardized DALYs rate of cirrhosis due to NASH in China ranked 185th from high to low, the age‑standardized DALYs rate of liver cancer due to NASH in China ranked 30th around the world. Thus, there might be underlying social and medical explanation. NAFLD-related liver cancer may also occur in non-cirrhotic livers. In a study from the Veterans Administration system, 13% of patients with liver cancer did not have cirrhosis, which was different from the traditional NAFLD-NASH-cirrhosis-hepatocellular carcinoma sequence 27, 28. Our findings suggested that liver cancer in NASH without cirrhosis should be paid attention to in future. Besides, coexisting conditions such as Hepatitis B Virus (HBV) infection and aflatoxin intake in China may accelerate the phenomenon 29, 30. Another possible explanation was that cirrhosis due to NASH may be underestimated caused by poor patient acceptability of liver biopsy during compensation and inaccuracy of noninvasive methods to diagnose NASH and cirrhosis 31. With an increasing health burden of NASH-related end-stage liver disease, there is an urgent need to develop accurate noninvasive methods for diagnosis of NAFLD and NASH and monitoring of fibrosis progression.

We observed gender disparity in cirrhosis and liver cancer burden due to NASH by year, age, and different SDI levels. Extensive evidence suggested that the prevalence of NAFLD/NASH in male was higher than that in female, especially female of fertile age 32. A large cross-sectional population-based survey in 39,151 Japanese subjects (health check-up) with the mean age of 45.8 years showed that the prevalence of NAFLD was 26% in Male and 12.7% in female 33. After menopause female display a similar or even a higher DALYs number of cirrhosis due to NASH compared to male after 70 years old, a finding supporting a protective effect of estrogens 34. Data in rats and human models suggested that high estrogen level is negatively associated with hepatic steatosis through changes in gene expression of molecules related to fat oxidation and lipogenesis 32. What's more, although the global prevalence of obesity in female (6%-15%) was higher than that in male (3%-11%), male had more visceral fat than female, which also explained the heavier disease burden of male than that of female 32, 35. Understanding the epidemiology of NAFLD/NASH is helpful to interpret gender disparity in the health burden of cirrhosis and liver cancer burden due to NASH. On the other hand, the role of gender in fibrosis progression in NAFLD/NASH is still multifaceted, although some studies have reported that the risk was significantly increased in male than in female 36. In addition, the burden of liver cancer due to NASH was higher in male than female also probably be due to the tumorigenic effect of androgens 37, 38.

The strengths of our study were the comprehensive estimations of burden of cirrhosis and liver cancer as measured with incidence, prevalence, death, and DALYs from 1990 to 2017, and the projection for 2018 towards 2050. It is worth-mentioning that DALYs, which were useful for quantifying and ranking disease burden, were thoroughly studied in our research. DALYs were generated by summing years of life lost due to premature death and years lived with disability. The years of life lost represent the years lost due to premature death; the years live with disability represents the years live with cirrhosis and liver cancer due to NASH. DALYs reflects the actual health status 10. As shown earlier, in our research, the global age-standardized prevalence rates of cirrhosis and liver cancer due to NASH increased from 1990 to 2017, while the global age-standardized DALYs rates of cirrhosis and liver cancer due to NASH were not increasing, which may be due to recent advances in early diagnosis and therapy of cirrhosis and liver cancer 20, 21 and could help decision makers understand the importance of paying attention to cirrhosis and liver cancer due to NASH.

However, there were several limitations of this study. First, our analysis shared the limitations of GBD estimates 11-16. The accuracy of the GBD estimates was limited by the quality and availability of original hospital and claims data, and the method of data processing may inevitably introduce bias. Second, cases due to NASH were defined only if NASH was listed as a specific etiology in the manuscript. Liver biopsy is still gold standard of NASH and fibrosis progression, with limitations including invasiveness, poor acceptability and sampling variability, which may cause underestimation of NASH and cirrhosis. Third, patients admitted to hospitals mostly had decompensated cirrhosis, thus number of compensated cirrhosis may be underestimated. Fourth, subgroup analyses by with or without alcoholic hepatitis and virus hepatitis were not performed. Notwithstanding the above limitations, the findings of this study could serve as an impetus for continued efforts toward eliminating cirrhosis and liver cancer due to NASH.

## Conclusion

In conclusion, the global, comparative study demonstrated the trend of health burden of cirrhosis and liver cancer due to NASH by age, gender, location and socioeconomic levels. Cirrhosis and liver cancer due to NASH was becoming a serious global public health problem and would keep worsening if without interventions. Male had a higher health burden compared with that of female. Geographic influences existed, while low SDI countries and low-middle SDI countries tended to bear more burden. These findings will hopefully raise awareness of cirrhosis and liver cancer due to NASH and reveal an urgent need for prevention of NASH.

## Supplementary Material

Supplementary figures and tables.

## Figures and Tables

**Table 1:** Global burden of cirrhosis and liver cancer due to NASH in 1990 and in 2017

	Cirrhosis due to NASH			Liver cancer due to NASH		
	1990	2017	Change (%)	1990	2017	Change (%)
**Incidence Number**						
Both	178,430 (162,931-195,848)	367,779 (334,461-403,729)	106.1	28,316 (24,859-32,303)	72,197 (64,602-79,915)	155.0
Female	72,218 (65,621-79,579)	155,446 (140,604-170,785)	115.2	11,489 (9,940-13,093)	25,966 (23,192-28,899)	126.0
Male	106,212 (96,992-116,227)	212,333 (193,932-232,724)	99.9	16,827 (14,381-19,793)	46,231 (40,859-51,763)	174.7
**Prevalence Number**						
Both	395,517,822 (379,947,260-411,747,556)	892,322,802 (858,624,902-927,954,380)	125.6	31,861 (27,921-36,296)	97,376 (86,789-108,049)	205.6
Female	156,736,936 (150,553,823-163,266,724)	362,528,343 (348,459,597-377,017,855)	131.3	12,264 (10,545-13,906)	31,002 (27,649-34,461)	152.8
Male	238,780,885 (229,292,934-248,544,047)	529,794,459 (509,933,144-550,960,010)	121.9	19,597 (16,757-23,020)	66,374 (58,436-74,563)	238.7
**Death Number**						
Both	61,880 (55,395-67,979)	118,030 (108,618-128,577)	90.7	28,528 (25,037-32,528)	66,875 (59,560-74,511)	134.4
Female	26,971 (23,761-29,716)	49,916 (45,639-55,865)	85.1	11,963 (10,389-13,638)	25,741 (22,883-28,696)	115.2
Male	34,910 (30,599-38,682)	68,113 (61,977-74,870)	95.1	16,566 (14,132-19,493)	41,134 (36,391-45,989)	148.3
**DALYs Number**						
Both	1,883,893 (1,693,315-2,064,605)	3,434,185 (3,153,991-3,743,299)	82.3	711,891 (624,591-816,722)	1,461,284 (1,304,838-1,623,665)	105.3
Female	758,250 (678,067-833,931)	1,328,968 (1,216,931-1,471,805)	75.3	270,922 (233,020-308,498)	505,289 (451,349-559,662)	86.5
Male	1,125,643 (996,675-1,240,126)	2,105,217 (1,924,086-2,313,409)	87.0	440,969 (374,405-521,592)	955,994 (842,200-1,073,864)	116.8
**Age-standardized Incidence Rate**						
Both	3.7 (3.4-4)	4.6 (4.2-5.1)	24.3	0.7 (0.6-0.8)	0.9 (0.8-1)	28.6
Female	3 (2.8-3.3)	3.8 (3.4-4.2)	26.7	0.5 (0.5-0.6)	0.6 (0.5-0.7)	20.0
Male	4.4 (4.1-4.8)	5.4 (5-5.9)	22.7	0.9 (0.8-1)	1.2 (1.1-1.4)	33.3
**Age-standardized Prevalence Rate**						
Both	8,287.2 (7,972.2-8,627.8)	11,060.5 (10,651.3-11,493.1)	33.5	0.8 (0.7-0.9)	1.2 (1.1-1.3)	50.0
Female	6,506.1 (6,250.6-6,776)	8,825.7 (8,481-9,179.1)	35.7	0.6 (0.5-0.6)	0.7 (0.6-0.8)	16.7
Male	10,133.7 (9,751.7-10,547.2)	13,352.6 (12,861-13,875)	31.8	1 (0.8-1.1)	1.7 (1.5-1.9)	70.0
**Age-standardized Death Rate**						
Both	1.5 (1.3-1.6)	1.5 (1.3-1.6)	0.0	0.7 (0.6-0.8)	0.8 (0.8-0.9)	14.3
Female	1.2 (1.1-1.4)	1.2 (1.1-1.3)	0.0	0.6 (0.5-0.6)	0.6 (0.5-0.7)	0.0
Male	1.7 (1.5-1.9)	1.8 (1.6-1.9)	5.9	0.9 (0.8-1.1)	1.1 (1-1.3)	22.2
**Age-standardized DALYs Rate**						
Both	42.4 (38.1-46.5)	41.8 (38.5-45.5)	-1.4	16.5 (14.5-18.9)	18 (16.1-20)	9.1
Female	33.5 (29.9-36.8)	31.6 (29-35)	-5.7	12 (10.4-13.7)	11.9 (10.6-13.2)	-0.8
Male	51.3 (45.5-56.7)	52.2 (47.8-57.3)	1.8	21.3 (18.2-25.2)	24.6 (21.8-27.6)	15.5

NASH: nonalcoholic steatohepatitis; DALYs: disability-adjusted life years.

**Table 2:**
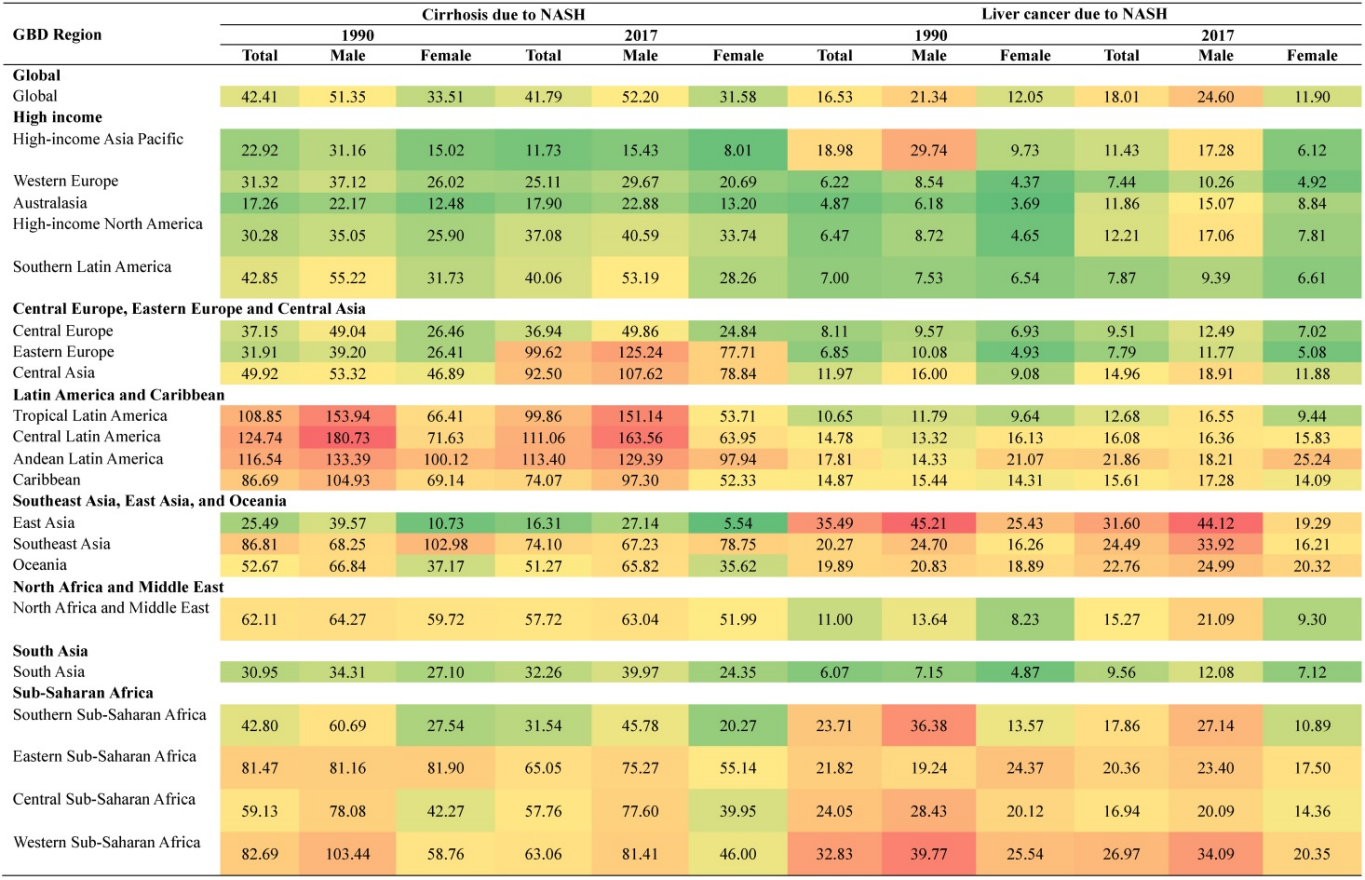
Age-standardized DALYs rate of NASH per 100 000 persons by GBD Region in 1990 and 2017

NASH: nonalcoholic steatohepatitis; DALYs: disability-adjusted life years.

## Abbreviations

NASH: Nonalcoholic Steatohepatitis
GBD: Global Burden of Disease
SDI: Socio-demographic Index
DALYs: disability-adjusted life years
NAFLD: Nonalcoholic fatty liver disease
TFU25: total fertility rate under the age of 25
EDU15+: education for those aged 15 and older
LDI: lag distributed income
ARIMA: Auto-Regressive Integrated Moving Average
UI: uncertainty interval
PM: particulate matter
HBV: Hepatitis B Virus
