# Supplementary material for: Age, Gender and Geographic Differences in Global Health Burden of Cirrhosis and Liver Cancer due to Nonalcoholic Steatohepatitis
Source: J Cancer. 2021 Mar 15;12(10):2855–65. doi: 10.7150/jca.52282 (PMC8040882; doi:10.7150/jca.52282)
Supplement: Supplementary file 1 — Supplementary figures and tables. [file jcav12p2855s1.pdf]

Supplementary materials

1.Supplementary Statistical Analysis Methods: The code applied to ARIMA model.

2. Supplementary Tables.

3.Supplementary figures.

Supplementary Statistical Analysis Methods:

Auto-regressive Integrated Moving Average model (ARIMA) was widely used in time series analysis. Auto.arima function in R language was used to establish ARIMA model. The code is as follows.

```
#
library(forecast)
data1 <- read.table(' data_to_predict.txt ',sep = " \t ",header = T)
data2 <- data1[-1]
data2 <- ts(data2,start = c(1990))
fit <- auto.arima(data2)
fit
plot1 <- plot(forecast(fit,h = 33))
plot1
predict1 <- forecast(fit,h = 33)
predict_mean <- print(predict1$mean)
```

Table S1 Global burden of cirrhosis due to NASH by country/territory in 2017

Country/ Territory	Incidence		Prevalence		Death		DALYs		Population
	Number	Age-standardized Rate	Number	Age-standardized Rate	Number	Age-standardized Rate	Number	Age-standardized Rate	
Afghanistan	1,572	9.4	3,039,759	15401.0	200	1.9	6,477	49.4	32,854,767
Albania	145	4.7	385,696	10912.7	22	0.5	636	16.2	2,766,162
Algeria	3,206	8.3	6,748,169	16794.0	375	1.2	10,446	29.6	40,463,698
American Samoa	3	6.3	11,989	23508.8	1	2.3	31	60.4	55,631
Andorra	4	3.7	8,274	7620.2	1	0.7	28	21.5	79,966
Angola	1,037	6.1	1,683,821	9424.2	352	3.0	11,740	83.0	28,202,299
Antigua and Barbuda	10	9.5	13,604	13280.2	2	2.4	67	63.3	88,957
Argentina	2,546	5.3	3,848,095	7949.4	644	1.2	16,817	33.4	44,265,909
Armenia	193	5.2	339,764	9164.2	91	2.2	2,013	49.9	3,027,231
Australia	697	2.4	2,620,548	8728.2	255	0.7	6,551	18.9	23,943,082
Austria	511	5.1	928,249	7755.7	171	1.1	4,515	31.3	8,793,125
Azerbaijan	638	6.1	1,080,407	10102.0	276	3.1	7,646	74.8	10,225,021
Bahrain	148	9.6	394,071	22833.0	13	1.4	444	35.2	1,470,373
Bangladesh	3,241	2.4	12,648,657	8636.6	1,478	1.2	44,055	32.3	156,981,002
Barbados	32	8.7	54,027	14029.1	8	1.8	224	50.0	295,884
Belarus	568	5.3	1,012,096	7884.3	271	1.8	8,659	61.7	9,491,040
Belgium	311	2.4	1,120,239	7543.7	150	0.7	3,710	20.4	11,319,411

Belize	41	11.5	50,632	14709.0	12	4.3	359	118.5	394,958
Benin	482	6.9	706,632	9275.5	89	1.8	2,873	49.6	11,585,301
Bermuda	7	8.4	14,249	15434.4	2	1.2	39	34.2	65,886
Bhutan	23	2.8	98,730	11088.4	9	1.5	285	38.5	957,410
Bolivia	1,506	15.0	1,228,013	11983.2	497	5.8	12,642	138.8	11,542,995
Bosnia and Herzegovina	214	5.2	601,222	12676.2	47	0.8	1,362	24.4	3,399,239
Botswana	84	4.1	222,475	10835.8	16	1.1	495	29.9	2,281,843
Brazil	22,124	9.4	26,670,918	11373.2	8,213	3.5	241,305	100.6	211,812,707
Brunei	12	2.4	37,364	8384.4	1	0.3	47	11.3	432,451
Bulgaria	410	4.9	836,767	7812.0	140	1.1	4,161	36.5	7,052,339
Burkina Faso	856	6.6	1,017,121	7337.7	125	1.4	3,930	36.7	21,121,966
Burundi	415	6.5	440,716	6284.0	111	2.6	3,528	67.4	10,905,413
Cambodia	943	7.7	1,687,861	11709.8	935	8.6	25,661	203.2	16,122,383
Cameroon	1,318	7.5	2,094,453	11059.4	322	2.6	10,493	72.3	27,769,738
Canada	1,066	2.0	3,291,538	6893.2	453	0.7	11,438	19.5	35,982,918
Cape Verde	28	5.5	54,553	10514.4	7	1.4	209	44.0	545,806
Central African Republic	149	4.8	232,112	6937.6	37	1.5	1,265	44.5	4,622,515
Chad	438	5.5	650,886	7624.7	102	1.7	3,344	49.2	15,222,086
Chile	1,475	7.4	2,097,081	9952.2	502	2.1	13,321	57.4	17,918,235
China	63,431	3.9	234,822,311	12828.1	10,725	0.5	324,591	15.9	1,412,480,390
Colombia	4,446	8.5	5,664,215	10673.3	569	1.1	14,667	27.1	50,606,157
Comoros	36	6.5	52,860	8874.7	7	1.5	196	38.4	718,322

Congo	190	5.1	316,902	8196.6	65	2.4	2,108	65.6	4,913,072
Costa Rica	633	12.4	552,466	10941.9	136	2.7	3,626	71.4	4,653,734
Cote d'Ivoire	1,039	6.3	1,578,752	8954.8	219	1.9	7,441	53.8	24,965,279
Croatia	246	5.0	554,368	8837.6	79	1.0	2,169	29.9	4,275,496
Cuba	1,206	8.5	1,830,008	12059.7	387	2.1	10,696	60.1	11,376,669
Cyprus	47	2.9	137,604	8493.8	12	0.7	295	16.6	1,262,632
Czech Republic	501	4.0	1,181,685	7634.4	151	0.8	4,524	27.1	10,592,732
Democratic Republic of the Congo	2,437	4.7	3,782,318	6935.5	653	1.7	21,841	49.2	80,884,671
Denmark	246	3.9	506,280	6742.3	88	0.9	2,403	26.5	5,732,278
Djibouti	63	7.4	81,021	8714.3	12	2.1	378	52.9	1,113,100
Dominica	7	8.5	9,875	12318.5	2	2.2	52	60.1	68,933
Dominican Republic	981	9.4	1,103,576	10787.5	290	3.2	7,506	78.5	10,451,750
Ecuador	2,586	16.2	3,035,437	18809.9	871	6.0	21,413	141.4	16,686,189
Egypt	10,684	13.7	18,219,416	21924.8	5,715	13.4	141,197	239.9	96,484,009
El Salvador	978	16.4	847,176	14186.4	249	4.4	6,889	121.3	6,086,947
Equatorial Guinea	61	7.2	99,981	11127.9	13	2.6	447	72.5	1,345,031
Eritrea	237	6.5	226,930	5508.1	70	3.0	2,338	78.0	5,859,329
Estonia	70	4.8	142,096	7653.1	30	1.5	960	50.8	1,314,543
Ethiopia	3,124	5.2	4,757,300	7182.1	894	2.3	26,445	56.2	102,883,938

Federated States of Micronesia	5	5.3	17,163	18815.4	2	2.2	51	59.6	103,926
Fiji	31	3.7	164,000	18647.3	7	1.0	227	26.4	906,939
Finland	304	4.9	537,499	7188.5	128	1.3	3,750	43.8	5,517,978
France	2,567	3.5	5,977,420	7043.5	949	0.8	24,305	23.8	65,712,599
Gabon	80	5.8	147,582	10312.6	31	2.8	960	77.7	1,702,720
Georgia	238	5.1	389,959	8175.1	113	2.1	3,237	63.1	3,691,322
Germany	4,964	5.2	9,120,835	7812.7	1,976	1.2	50,532	35.2	83,294,524
Ghana	1,397	6.3	2,083,088	8733.4	302	1.9	9,679	50.5	30,205,292
Greece	445	3.6	1,332,678	9083.5	132	0.6	3,104	17.2	10,402,319
Greenland	2	2.1	4,600	7004.7	1	0.9	22	27.7	56,155
Grenada	11	8.9	16,198	12631.3	4	2.6	96	72.3	110,879
Guam	8	4.8	31,842	17963.5	4	2.2	119	63.4	167,837
Guatemala	2,703	18.1	2,318,994	16409.7	707	6.3	22,164	182.0	16,924,208
Guinea	434	5.9	629,785	8092.1	96	1.7	2,927	46.7	11,819,987
Guinea-Bissau	81	7.2	128,517	10408.2	19	2.5	652	70.3	1,855,540
Guyana	81	11.8	87,206	12310.9	34	5.1	1,041	147.0	742,302
Haiti	907	9.2	1,044,782	10901.6	263	3.8	7,788	99.2	11,824,835
Honduras	1,470	18.4	1,326,507	17184.0	394	6.4	11,522	173.3	9,498,815
Hungary	609	5.3	1,230,037	8606.6	233	1.4	6,844	43.6	9,727,364
Iceland	27	7.2	39,068	9530.4	3	0.7	111	24.0	337,471
India	25,214	2.1	98,235,850	7526.4	11,290	1.0	355,010	28.6	1,380,560,359
Indonesia	13,241	6.1	44,245,183	16858.0	8,260	4.2	219,144	93.8	258,134,673

Iran	4,086	4.8	17,971,815	20500.1	747	1.1	19,695	26.1	82,176,111
Iraq	2,059	6.8	6,834,281	20099.8	142	0.6	5,056	18.2	43,304,442
Ireland	187	3.4	576,287	9759.5	41	0.6	1,198	18.2	4,860,552
Israel	389	4.3	1,372,384	14540.2	84	0.7	1,929	18.7	8,949,198
Italy	2,119	3.0	10,723,617	12291.6	699	0.5	14,913	12.9	60,597,480
Jamaica	238	8.0	395,858	13368.6	39	1.3	1,078	37.0	2,779,264
Japan	2,736	1.7	12,475,423	6516.1	1,165	0.3	24,000	9.5	128,363,257
Jordan	884	10.5	2,035,242	22342.9	69	1.3	2,171	32.0	10,648,463
Kazakhstan	1,129	6.1	1,395,063	7619.5	551	3.1	17,447	92.4	17,904,334
Kenya	2,490	7.6	2,812,594	8114.9	1,043	4.8	31,853	123.6	48,326,776
Kiribati	6	6.1	19,513	20358.8	2	2.9	66	77.7	118,241
Kuwait	411	9.1	1,123,563	23864.7	28	1.1	1,021	30.6	4,262,200
Kyrgyzstan	369	6.4	476,452	8572.7	165	3.3	5,597	103.0	6,368,576
Laos	307	6.1	600,973	10254.1	141	3.3	4,223	84.1	6,970,176
Latvia	104	4.8	221,013	7853.4	48	1.5	1,509	52.8	1,945,427
Lebanon	660	8.7	1,439,136	18433.1	70	1.2	1,934	30.2	8,511,980
Lesotho	73	4.4	152,119	9196.8	17	1.3	530	38.7	1,947,578
Liberia	221	7.1	353,009	10668.2	49	2.4	1,577	63.3	4,722,638
Libya	750	11.8	1,511,427	22495.0	94	2.2	2,779	52.3	6,908,740
Lithuania	181	5.9	318,127	7776.4	99	2.2	3,222	78.6	2,847,647
Luxembourg	38	5.6	62,967	8257.7	10	1.1	268	30.5	590,547
Macedonia	129	4.9	283,199	9738.2	20	0.6	628	19.6	2,174,828
Madagascar	897	5.8	1,031,258	5931.8	191	1.8	6,202	46.5	26,108,785
Malawi	808	7.5	933,956	8117.3	183	2.4	5,366	62.6	17,191,217
Malaysia	1,328	4.8	6,154,515	19957.1	394	1.6	10,344	37.3	30,639,828

Maldives	19	4.9	85,082	18165.3	2	0.8	61	18.7	458,601
Mali	774	6.7	1,233,664	9964.2	102	1.2	3,311	33.0	20,253,727
Malta	16	3.0	61,297	10253.9	5	0.7	136	19.2	434,472
Marshall Islands	3	5.9	9,649	20192.6	1	2.9	34	78.9	56,311
Mauritania	184	7.1	359,355	12958.2	35	1.7	1,058	45.9	3,913,922
Mauritius	82	5.3	216,980	13850.3	27	1.6	786	45.5	1,272,112
Mexico	24,902	19.4	19,866,665	15745.7	6,547	5.6	187,097	152.2	126,569,720
Moldova	501	10.9	407,542	8324.2	364	6.5	10,562	192.5	3,721,820
Mongolia	197	6.5	221,408	7359.9	105	4.8	3,252	118.8	3,251,426
Montenegro	29	3.9	70,557	8540.8	4	0.4	126	13.9	626,318
Morocco	2,978	8.8	6,312,316	17578.5	427	1.4	11,476	34.3	35,488,915
Mozambique	1,137	6.8	1,104,794	5938.0	157	1.4	5,127	36.9	30,035,304
Myanmar	3,380	7.0	7,336,426	14000.3	1,935	4.0	61,349	117.9	52,795,004
Namibia	61	3.2	152,240	7933.7	14	1.0	443	27.6	2,353,503
Nepal	538	2.2	2,488,859	9488.9	283	1.3	8,305	34.5	29,891,524
Netherlands	648	3.4	1,988,210	8821.4	178	0.5	4,100	14.3	17,029,098
New Zealand	117	2.3	460,409	8373.3	36	0.5	866	12.8	4,448,375
Nicaragua	976	16.4	955,771	16883.7	204	4.3	6,106	121.7	6,396,570
Niger	663	6.0	943,411	7967.3	76	1.0	2,555	27.5	21,375,946
Nigeria	7,124	5.6	9,550,957	7058.6	2,734	3.1	85,949	80.9	206,087,947
North Korea	1,013	3.6	2,944,609	9457.3	242	0.8	7,343	22.1	25,716,644
Northern Mariana Islands	2	4.2	9,656	17980.7	1	1.9	29	48.0	44,878

Norway	166	2.8	487,542	7269.5	36	0.4	946	11.9	5,263,178
Oman	435	9.9	1,092,361	23172.3	27	1.4	956	36.1	4,535,815
Pakistan	4,089	2.8	18,265,042	10994.1	2,879	2.6	90,258	64.6	214,287,443
Palestine	279	8.3	692,933	18665.1	29	1.3	855	31.3	4,852,097
Panama	360	9.0	533,249	13362.1	64	1.6	1,642	41.1	3,921,083
Papua New Guinea	266	3.8	1,092,795	15589.7	88	1.6	3,292	50.7	9,227,551
Paraguay	570	8.9	593,015	9498.5	137	2.5	3,939	68.6	6,931,175
Peru	3,872	11.8	2,967,771	9160.7	1,122	3.7	28,587	92.4	33,219,612
Philippines	2,937	3.8	9,626,347	10572.0	1,152	1.6	33,224	40.4	103,470,619
Poland	2,296	5.0	4,032,002	7491.6	552	0.9	17,790	31.1	38,393,061
Portugal	464	3.6	1,648,675	10881.8	219	1.1	5,626	31.5	10,681,712
Puerto Rico	498	10.9	707,768	14367.4	209	3.2	5,000	85.7	3,665,882
Qatar	341	11.8	800,503	25229.2	19	2.3	712	52.2	2,747,311
Romania	1,655	7.4	2,233,334	7994.0	743	2.2	20,476	65.9	19,433,766
Russian Federation	10,993	6.6	16,777,806	8697.1	5,708	2.7	189,902	93.7	146,189,867
Rwanda	539	6.3	486,698	5391.2	146	2.5	4,430	64.2	12,554,172
Saint Lucia	20	9.9	27,839	13640.8	6	2.8	166	77.2	176,474
Saint Vincent and the Grenadines	14	11.4	19,545	15304.7	4	3.0	121	89.3	114,112
Samoa	9	5.6	37,119	22871.5	3	2.2	85	55.6	198,918
Sao Tome and Principe	12	7.7	16,052	10492.1	5	5.0	171	135.4	200,206

Saudi Arabia	3,625	10.5	8,403,186	23596.1	402	3.0	12,033	61.2	34,444,054
Senegal	595	6.1	1,065,215	10273.1	91	1.2	2,872	33.7	14,688,034
Serbia	376	3.6	1,132,153	9177.9	98	0.7	2,794	20.5	8,874,098
Seychelles	9	7.8	21,142	18446.0	4	3.4	102	86.7	100,907
Sierra Leone	331	6.4	481,194	8850.5	67	1.8	2,173	51.4	7,829,749
Singapore	58	0.8	627,866	9087.9	7	0.1	223	3.1	5,568,481
Slovakia	435	6.7	540,651	7267.3	109	1.3	3,463	42.1	5,419,210
Slovenia	129	5.3	276,218	9111.9	42	1.1	1,201	34.5	2,068,856
Solomon Islands	21	4.6	78,665	16504.7	7	1.9	235	55.2	637,607
Somalia	683	7.1	799,005	7545.5	171	2.7	5,358	68.8	16,880,386
South Africa	2,079	3.9	6,571,175	12125.8	486	1.1	15,014	30.4	54,952,860
South Korea	2,256	3.5	5,854,263	8239.0	490	0.6	14,893	17.4	52,670,741
South Sudan	427	7.7	527,555	8557.3	102	2.6	3,230	68.3	9,941,010
Spain	2,102	3.9	6,783,500	10488.9	947	1.0	20,837	26.3	46,389,213
Sri Lanka	1,111	4.7	3,078,639	12769.3	368	1.5	9,899	38.8	21,596,437
Sudan	2,545	9.8	4,915,498	17427.8	334	2.0	9,276	45.6	40,255,551
Suriname	64	10.8	74,584	12369.5	24	4.1	670	108.0	572,463
Swaziland	43	5.0	104,560	11932.4	11	1.8	364	52.7	1,124,436
Sweden	402	3.6	1,078,081	8193.9	117	0.6	2,794	17.2	10,044,996
Switzerland	284	2.7	795,418	6871.7	86	0.5	2,120	15.0	8,593,087
Syria	1,317	9.0	3,209,396	20460.9	204	1.7	5,811	41.0	18,131,194
Taiwan	1,471	5.5	3,972,308	12338.3	468	1.2	13,181	36.4	23,583,126
Tajikistan	366	4.9	535,463	7351.0	107	1.8	3,758	54.9	9,243,717
Tanzania	2,226	6.7	2,803,153	7717.3	449	1.9	13,214	47.3	53,973,136

Thailand	6,096	6.6	14,555,968	15961.2	2,385	2.4	69,391	68.9	70,626,063
The Bahamas	37	9.0	48,877	11917.6	10	2.6	314	74.0	375,441
The Gambia	88	6.4	137,872	9464.9	19	1.9	590	53.5	2,132,510
Timor-Leste	46	5.4	111,629	11589.2	20	2.6	554	64.6	1,287,486
Togo	294	5.7	458,811	8295.8	46	1.2	1,566	34.7	7,516,030
Tonga	6	6.8	20,970	23301.8	3	3.6	77	90.5	102,838
Trinidad and Tobago	158	9.6	233,784	14042.9	40	2.2	1,188	65.3	1,391,805
Tunisia	1,593	12.8	2,107,015	16616.1	149	1.3	3,886	31.2	11,442,183
Turkey	4,998	5.7	16,291,752	18361.8	709	0.8	19,201	21.5	80,456,851
Turkmenistan	363	7.5	414,419	8883.4	189	4.2	6,849	140.9	4,976,883
Uganda	1,341	6.4	1,344,050	5669.5	284	2.0	8,888	53.1	39,078,382
Ukraine	3,545	7.1	5,389,565	8673.5	2,127	3.3	76,119	124.1	44,689,084
United Arab Emirates	1,239	10.6	2,855,520	22973.0	62	1.7	2,453	43.2	9,734,100
United Kingdom	2,688	3.6	7,545,042	8821.1	997	0.9	29,002	30.2	66,635,515
United States	13,025	2.9	40,973,831	10000.1	6,780	1.3	187,700	39.2	324,839,024
Uruguay	208	5.3	479,806	11627.8	50	1.0	1,196	25.5	3,421,532
Uzbekistan	2,050	6.9	2,683,266	9191.0	904	3.6	30,623	107.5	32,236,895
Vanuatu	10	4.7	36,309	16367.1	5	2.8	163	81.2	287,568
Venezuela	4,044	12.6	5,328,912	16985.1	647	2.2	19,224	63.2	30,831,741
Vietnam	6,684	6.8	11,092,691	10684.4	1,806	2.0	47,172	47.4	96,140,732
Virgin Islands, U.S.	13	10.8	17,661	12788.7	7	3.6	165	95.4	104,952

Yemen	1,459	7.7	3,257,677	15574.3	160	1.3	4,728	32.2	30,449,159
Zambia	724	7.1	864,663	7688.6	226	3.5	7,012	87.8	17,364,088
Zimbabwe	454	4.3	1,008,010	9539.4	98	1.4	3,044	37.3	14,713,754

NASH: nonalcoholic steatohepatitis; DALYs: disability-adjusted life years.

Table S2 Global burden of liver cancer due to NASH by country/territory in 2017

Country/ Territory	Incidence		Prevalence		Death		DALYs		Population
	Number	Age-standardized Rate	Number	Age-standardized Rate	Number	Age-standardized Rate	Number	Age-standardized Rate	
Afghanistan	55	0.6	59	0.6	56	0.6	1,472	12.9	32,854,767
Albania	26	0.6	26	0.6	29	0.7	573	13.6	2,766,162
Algeria	73	0.2	72	0.2	79	0.3	1,648	5.0	40,463,698
American Samoa	1	1.6	1	1.5	1	1.7	16	35.4	55,631
Andorra	0	0.2	0	0.3	0	0.2	5	3.9	79,966
Angola	86	0.9	97	0.9	87	0.9	2,362	20.2	28,202,299
Antigua and Barbuda	1	0.8	1	0.7	1	0.9	17	16.5	88,957
Argentina	176	0.3	167	0.3	195	0.4	3,737	7.1	44,265,909
Armenia	30	0.7	27	0.7	33	0.8	610	14.7	3,027,231
Australia	268	0.7	353	0.9	249	0.6	4,536	11.8	23,943,082
Austria	69	0.4	111	0.7	56	0.3	972	5.8	8,793,125
Azerbaijan	52	0.6	55	0.6	54	0.7	1,285	13.6	10,225,021
Bahrain	3	0.4	3	0.4	3	0.4	76	8.1	1,470,373

Bangladesh	266	0.2	284	0.2	279	0.2	6,685	5.2	156,981,002
Barbados	3	0.6	3	0.6	3	0.7	62	13.1	295,884
Belarus	36	0.2	35	0.2	39	0.2	796	5.1	9,491,040
Belgium	77	0.3	83	0.4	77	0.3	1,241	5.8	11,319,411
Belize	3	1.1	3	1.1	3	1.2	70	25.6	394,958
Benin	72	1.6	77	1.6	74	1.7	1,860	36.2	11,585,301
Bermuda	1	0.5	1	0.4	1	0.5	12	9.4	65,886
Bhutan	2	0.4	3	0.4	3	0.5	59	9.3	957,410
Bolivia	111	1.4	105	1.2	122	1.5	2,412	28.0	11,542,995
Bosnia and Herzegovina	46	0.8	43	0.7	51	0.8	971	16.0	3,399,239
Botswana	10	0.8	11	0.8	11	0.9	263	17.8	2,281,843
Brazil	1,277	0.6	1,303	0.6	1,369	0.6	29,542	12.8	211,812,707
Brunei	2	0.7	3	0.8	2	0.8	56	16.7	432,451
Bulgaria	64	0.4	61	0.4	70	0.5	1,386	10.1	7,052,339
Burkina Faso	131	1.5	142	1.5	130	1.6	3,403	34.7	21,121,966
Burundi	24	0.6	27	0.6	24	0.7	680	14.4	10,905,413
Cambodia	67	0.6	72	0.6	70	0.7	1,685	14.2	16,122,383
Cameroon	200	1.8	216	1.8	203	1.9	5,356	41.6	27,769,738
Canada	479	0.7	632	1.0	423	0.6	7,616	11.9	35,982,918
Cape Verde	5	1.1	5	1.1	6	1.2	117	25.8	545,806
Central African Republic	15	0.7	16	0.7	15	0.8	414	17.7	4,622,515
Chad	73	1.4	77	1.3	75	1.5	1,893	31.7	15,222,086

Chile	118	0.5	110	0.5	131	0.6	2,376	10.3	17,918,235
China	31,748	1.7	46,499	2.3	27,650	1.5	621,923	31.2	1,412,480,390
Colombia	272	0.5	257	0.5	304	0.6	5,696	10.6	50,606,157
Comoros	3	0.8	4	0.8	3	0.8	87	18.0	718,322
Congo	19	0.8	21	0.8	19	0.9	508	18.9	4,913,072
Costa Rica	38	0.8	37	0.7	42	0.9	819	16.7	4,653,734
Cote d'Ivoire	89	0.9	99	0.9	88	1.0	2,400	21.0	24,965,279
Croatia	58	0.6	72	0.8	55	0.6	940	10.8	4,275,496
Cuba	121	0.6	114	0.6	135	0.7	2,487	13.5	11,376,669
Cyprus	6	0.3	7	0.4	6	0.3	115	6.1	1,262,632
Czech Republic	77	0.4	75	0.4	80	0.4	1,411	6.9	10,592,732
Democratic Republic of the Congo	211	0.7	233	0.7	216	0.7	5,621	15.5	80,884,671
Denmark	35	0.3	43	0.4	33	0.3	583	5.4	5,732,278
Djibouti	5	0.9	6	0.9	5	1.0	141	22.2	1,113,100
Dominica	1	0.8	1	0.8	1	0.9	16	17.3	68,933
Dominican Republic	83	0.9	83	0.9	90	1.0	1,901	20.4	10,451,750
Ecuador	192	1.3	172	1.2	218	1.5	3,781	25.9	16,686,189
Egypt	1,153	1.9	1,345	2.0	1,138	2.0	32,170	47.5	96,484,009
El Salvador	51	0.9	49	0.9	56	1.0	1,105	19.5	6,086,947
Equatorial Guinea	5	1.2	6	1.2	5	1.3	139	27.5	1,345,031

Eritrea	21	0.9	24	1.0	21	1.0	617	23.0	5,859,329
Estonia	8	0.3	7	0.3	9	0.3	151	6.1	1,314,543
Ethiopia	182	0.5	198	0.5	186	0.5	4,821	10.9	102,883,938
Federated States of Micronesia	1	1.4	1	1.3	1	1.5	23	30.8	103,926
Fiji	7	1.0	7	0.9	7	1.0	168	21.7	906,939
Finland	59	0.5	107	0.9	44	0.3	706	6.1	5,517,978
France	702	0.5	754	0.6	712	0.5	11,861	9.6	65,712,599
Gabon	9	0.9	10	0.9	9	0.9	228	20.5	1,702,720
Georgia	31	0.5	30	0.5	34	0.6	701	12.4	3,691,322
Germany	1,129	0.6	1,786	1.0	928	0.5	15,698	9.0	83,294,524
Ghana	264	1.7	287	1.7	260	1.8	7,074	39.8	30,205,292
Greece	127	0.5	112	0.5	140	0.5	1,975	8.4	10,402,319
Greenland	0	0.7	1	0.7	1	0.8	12	16.2	56,155
Grenada	1	0.7	1	0.7	1	0.8	23	15.6	110,879
Guam	2	1.3	3	1.4	2	1.4	58	31.1	167,837
Guatemala	216	2.0	217	2.0	230	2.2	5,106	44.9	16,924,208
Guinea	185	3.4	196	3.4	189	3.7	4,811	81.5	11,819,987
Guinea-Bissau	13	1.9	15	1.9	13	2.0	369	45.4	1,855,540
Guyana	4	0.7	4	0.6	4	0.7	96	15.0	742,302
Haiti	44	0.7	46	0.7	46	0.8	1,098	15.9	11,824,835
Honduras	19	0.3	20	0.3	20	0.3	446	7.2	9,498,815
Hungary	72	0.4	69	0.4	79	0.4	1,523	8.3	9,727,364

Iceland	2	0.3	3	0.5	1	0.3	26	5.0	337,471
India	4,682	0.5	4,829	0.4	4,958	0.5	112,486	10.1	1,380,560,359
Indonesia	1,425	0.7	1,545	0.7	1,472	0.8	36,388	16.2	258,134,673
Iran	481	0.7	465	0.7	528	0.8	10,447	15.2	82,176,111
Iraq	84	0.4	89	0.4	88	0.4	2,048	8.5	43,304,442
Ireland	24	0.3	29	0.4	22	0.3	377	5.3	4,860,552
Israel	33	0.3	31	0.3	37	0.3	624	5.7	8,949,198
Italy	663	0.5	950	0.7	587	0.4	9,128	6.8	60,597,480
Jamaica	15	0.5	15	0.5	16	0.6	332	11.5	2,779,264
Japan	2,633	0.7	5,068	1.4	1,988	0.5	28,180	8.4	128,363,257
Jordan	18	0.4	18	0.3	19	0.4	415	7.1	10,648,463
Kazakhstan	65	0.4	68	0.4	67	0.4	1,587	9.0	17,904,334
Kenya	155	0.7	172	0.8	156	0.8	4,203	17.8	48,326,776
Kiribati	1	1.4	1	1.3	1	1.5	24	32.3	118,241
Kuwait	13	0.6	14	0.6	14	0.7	303	12.5	4,262,200
Kyrgyzstan	20	0.5	22	0.5	21	0.5	522	11.4	6,368,576
Laos	31	0.8	33	0.8	32	0.9	784	17.7	6,970,176
Latvia	12	0.3	11	0.3	13	0.3	247	6.7	1,945,427
Lebanon	20	0.3	22	0.4	20	0.4	430	7.0	8,511,980
Lesotho	13	1.1	14	1.1	13	1.2	344	26.2	1,947,578
Liberia	31	1.7	33	1.6	32	1.8	817	37.8	4,722,638
Libya	36	0.8	39	0.8	37	0.9	919	18.8	6,908,740
Lithuania	16	0.3	15	0.3	18	0.3	333	6.2	2,847,647
Luxembourg	4	0.5	6	0.6	4	0.4	65	7.0	590,547
Macedonia	22	0.6	22	0.6	23	0.7	500	14.8	2,174,828

Madagascar	60	0.6	69	0.6	60	0.6	1,726	14.3	26,108,785
Malawi	45	0.6	49	0.6	47	0.6	1,216	14.8	17,191,217
Malaysia	189	0.8	194	0.8	201	0.9	4,343	16.8	30,639,828
Maldives	1	0.5	1	0.5	1	0.5	31	10.4	458,601
Mali	286	3.5	319	3.6	288	3.7	7,749	83.3	20,253,727
Malta	2	0.2	2	0.3	2	0.2	37	4.3	434,472
Marshall Islands	1	1.7	1	1.7	1	1.9	14	40.2	56,311
Mauritania	31	1.6	33	1.6	32	1.7	753	35.7	3,913,922
Mauritius	7	0.4	7	0.4	7	0.5	146	8.8	1,272,112
Mexico	867	0.8	841	0.7	950	0.9	18,967	16.5	126,569,720
Moldova	41	0.7	44	0.8	43	0.8	1,013	17.9	3,721,820
Mongolia	131	7.0	140	6.6	138	7.8	3,262	148.2	3,251,426
Montenegro	5	0.5	5	0.5	6	0.5	105	10.6	626,318
Morocco	63	0.2	60	0.2	69	0.2	1,365	4.4	35,488,915
Mozambique	326	3.4	342	3.2	342	3.8	8,295	75.1	30,035,304
Myanmar	321	0.7	339	0.7	337	0.8	7,913	17.0	52,795,004
Namibia	4	0.3	4	0.3	4	0.3	104	7.0	2,353,503
Nepal	71	0.3	73	0.3	75	0.4	1,711	7.6	29,891,524
Netherlands	173	0.5	224	0.7	152	0.4	2,527	7.9	17,029,098
New Zealand	48	0.7	67	1.0	45	0.6	850	12.0	4,448,375
Nicaragua	46	1.0	46	1.0	49	1.1	1,068	22.7	6,396,570
Niger	142	2.1	155	2.0	143	2.2	3,766	46.4	21,375,946
Nigeria	327	0.4	331	0.4	348	0.5	7,734	9.2	206,087,947
North Korea	482	1.5	525	1.6	498	1.6	12,332	38.1	25,716,644

Northern Mariana Islands	0	0.9	1	0.9	0	1.0	10	18.9	44,878
Norway	20	0.2	29	0.3	17	0.2	297	3.4	5,263,178
Oman	11	0.6	12	0.6	11	0.6	285	12.9	4,535,815
Pakistan	446	0.4	504	0.4	440	0.4	12,390	9.6	214,287,443
Palestine	15	0.7	15	0.6	16	0.8	350	14.2	4,852,097
Panama	21	0.5	20	0.5	23	0.6	457	11.6	3,921,083
Papua New Guinea	35	0.8	41	0.8	33	0.9	1,025	20.1	9,227,551
Paraguay	17	0.3	17	0.3	18	0.4	380	7.1	6,931,175
Peru	270	0.9	249	0.8	302	1.0	5,556	18.2	33,219,612
Philippines	664	1.0	727	1.0	678	1.0	17,351	22.4	103,470,619
Poland	193	0.3	179	0.3	216	0.3	3,917	5.8	38,393,061
Portugal	88	0.4	86	0.4	96	0.4	1,653	7.7	10,681,712
Puerto Rico	55	0.8	51	0.8	61	0.8	1,069	16.0	3,665,882
Qatar	8	1.3	10	1.3	8	1.5	218	26.9	2,747,311
Romania	260	0.7	250	0.7	285	0.7	5,650	16.1	19,433,766
Russian Federation	813	0.3	822	0.4	871	0.4	18,399	8.1	146,189,867
Rwanda	36	0.6	40	0.6	37	0.7	964	15.2	12,554,172
Saint Lucia	1	0.6	1	0.5	1	0.6	26	12.3	176,474
Saint Vincent and the Grenadines	1	0.9	1	0.8	1	0.9	27	19.6	114,112

Samoa	1	0.6	1	0.6	1	0.6	19	13.6	198,918
Sao Tome and Principe	0	0.2	0	0.2	0	0.2	6	5.0	200,206
Saudi Arabia	108	0.9	118	0.8	111	1.0	2,678	17.7	34,444,054
Senegal	97	1.4	101	1.4	101	1.5	2,416	31.4	14,688,034
Serbia	78	0.5	73	0.5	85	0.5	1,608	10.2	8,874,098
Seychelles	1	0.7	1	0.7	1	0.7	18	15.9	100,907
Sierra Leone	46	1.4	48	1.3	47	1.5	1,201	31.5	7,829,749
Singapore	51	0.8	103	1.5	37	0.6	672	9.9	5,568,481
Slovakia	38	0.4	44	0.5	37	0.4	727	8.0	5,419,210
Slovenia	20	0.5	19	0.5	21	0.5	371	9.1	2,068,856
Solomon Islands	3	1.1	4	1.1	3	1.2	88	25.2	637,607
Somalia	49	0.8	56	0.8	49	0.8	1,406	19.2	16,880,386
South Africa	271	0.6	288	0.6	280	0.7	6,926	14.7	54,952,860
South Korea	1,275	1.5	2,598	3.0	909	1.1	17,828	20.6	52,670,741
South Sudan	35	1.0	40	1.0	36	1.0	999	23.5	9,941,010
Spain	397	0.4	583	0.7	340	0.3	5,602	6.4	46,389,213
Sri Lanka	71	0.3	74	0.3	75	0.3	1,563	6.2	21,596,437
Sudan	81	0.5	81	0.5	87	0.5	1,891	10.4	40,255,551
Suriname	6	1.0	6	0.9	6	1.1	128	21.7	572,463
Swaziland	8	1.5	9	1.5	9	1.6	246	37.4	1,124,436
Sweden	72	0.4	85	0.5	72	0.3	1,189	6.3	10,044,996
Switzerland	69	0.4	112	0.7	55	0.3	947	5.9	8,593,087
Syria	54	0.5	55	0.4	57	0.5	1,286	9.8	18,131,194

Taiwan	1,116	2.9	1,909	5.0	899	2.4	17,436	46.0	23,583,126
Tajikistan	21	0.4	24	0.4	20	0.4	653	9.7	9,243,717
Tanzania	179	0.8	193	0.8	184	0.8	4,671	17.8	53,973,136
Thailand	2,135	2.2	2,260	2.3	2,254	2.3	49,128	49.2	70,626,063
The Bahamas	2	0.7	3	0.7	3	0.7	58	15.2	375,441
The Gambia	36	3.8	39	3.9	37	4.1	975	93.9	2,132,510
Timor-Leste	6	0.7	6	0.7	6	0.8	132	16.1	1,287,486
Togo	39	1.2	44	1.2	40	1.3	1,067	27.5	7,516,030
Tonga	3	3.2	3	3.3	3	3.5	63	76.5	102,838
Trinidad and Tobago	9	0.5	9	0.5	9	0.5	192	10.7	1,391,805
Tunisia	20	0.2	19	0.2	21	0.2	410	3.4	11,442,183
Turkey	339	0.4	332	0.4	369	0.4	7,352	8.4	80,456,851
Turkmenistan	16	0.4	18	0.4	16	0.4	431	10.2	4,976,883
Uganda	123	0.9	136	0.9	126	1.0	3,401	22.0	39,078,382
Ukraine	235	0.3	256	0.3	238	0.3	5,055	7.0	44,689,084
United Arab Emirates	23	0.7	29	0.7	22	0.8	707	16.5	9,734,100
United Kingdom	509	0.4	740	0.6	434	0.3	7,007	5.9	66,635,515
United States	3,976	0.7	6,447	1.2	3,277	0.6	64,576	12.2	324,839,024
Uruguay	13	0.2	12	0.2	14	0.3	267	5.3	3,421,532
Uzbekistan	93	0.4	105	0.4	94	0.5	2,500	10.4	32,236,895
Vanuatu	2	1.6	3	1.5	3	1.7	66	37.0	287,568
Venezuela	173	0.6	174	0.6	186	0.7	3,931	13.9	30,831,741

Vietnam	1,207	1.3	1,315	1.4	1,255	1.4	30,213	31.6	96,140,732
Virgin Islands, U.S.	1	0.6	1	0.6	1	0.7	25	13.0	104,952
Yemen	36	0.3	37	0.3	37	0.3	865	6.6	30,449,159
Zambia	65	1.0	72	1.0	66	1.1	1,810	24.8	17,364,088
Zimbabwe	104	1.6	113	1.5	105	1.7	2,828	36.9	14,713,754

NASH: nonalcoholic steatohepatitis; DALYs: disability-adjusted life years.

Table S3 SDI groupings by country/territory, based on 2017 values

Country/ Territory	SDI	SDI group
Afghanistan	0.290255	Low SDI
Albania	0.684614	Middle SDI
Algeria	0.695849	Middle SDI
American Samoa	0.70186	High-middle SDI
Andorra	0.901838	High SDI
Angola	0.460536	Low-middle SDI
Antigua and Barbuda	0.715131	High-middle SDI
Argentina	0.710151	High-middle SDI
Armenia	0.702021	High-middle SDI
Australia	0.873188	High SDI
Austria	0.866029	High SDI
Azerbaijan	0.70117	High-middle SDI
Bahrain	0.712259	High-middle SDI
Bangladesh	0.457989	Low SDI
Barbados	0.739423	High-middle SDI
Belarus	0.772665	High-middle SDI
Belgium	0.886479	High SDI
Belize	0.602244	Low-middle SDI
Benin	0.373375	Low SDI
Bermuda	0.805453	High-middle SDI
Bhutan	0.569908	Low-middle SDI
Bolivia	0.587409	Low-middle SDI
Bosnia and Herzegovina	0.71261	High-middle SDI
Botswana	0.663238	Middle SDI
Brazil	0.663312	Middle SDI
Brunei	0.856241	High SDI
Bulgaria	0.791737	High-middle SDI
Burkina Faso	0.283938	Low SDI
Burundi	0.309706	Low SDI
Cambodia	0.481619	Low-middle SDI
Cameroon	0.482039	Low-middle SDI
Canada	0.882086	High SDI
Cape Verde	0.549086	Low-middle SDI
Central African Republic	0.334449	Low SDI
Chad	0.252902	Low SDI
Chile	0.748081	High-middle SDI
China	0.707319	High-middle SDI
Colombia	0.633692	Middle SDI
Comoros	0.43429	Low SDI
Congo	0.57413	Low-middle SDI
Costa Rica	0.66213	Middle SDI

Cote d'Ivoire	0.41214	Low SDI
Croatia	0.824845	High SDI
Cuba	0.687668	Middle SDI
Cyprus	0.864573	High SDI
Czech Republic	0.85098	High SDI
Democratic Republic of the Congo	0.364453	Low SDI
Denmark	0.917864	High SDI
Djibouti	0.48475	Low-middle SDI
Dominica	0.686587	Middle SDI
Dominican Republic	0.592641	Low-middle SDI
Ecuador	0.635567	Middle SDI
Egypt	0.604308	Low-middle SDI
El Salvador	0.593095	Low-middle SDI
Equatorial Guinea	0.625223	Middle SDI
Eritrea	0.408791	Low SDI
Estonia	0.857709	High SDI
Ethiopia	0.334181	Low SDI
Federated States of Micronesia	0.575252	Low-middle SDI
Fiji	0.641436	Middle SDI
Finland	0.892872	High SDI
France	0.864667	High SDI
Gabon	0.650559	Middle SDI
Georgia	0.699719	High-middle SDI
Germany	0.869902	High SDI
Ghana	0.536973	Low-middle SDI
Greece	0.816994	High SDI
Greenland	0.760075	High-middle SDI
Grenada	0.640418	Middle SDI
Guam	0.794193	High-middle SDI
Guatemala	0.524214	Low-middle SDI
Guinea	0.324711	Low SDI
Guinea-Bissau	0.348987	Low SDI
Guyana	0.583747	Low-middle SDI
Haiti	0.441666	Low SDI
Honduras	0.51234	Low-middle SDI
Hungary	0.816804	High-middle SDI
Iceland	0.907023	High SDI
India	0.550242	Low-middle SDI
Indonesia	0.647611	Middle SDI
Iran	0.700087	High-middle SDI
Iraq	0.584824	Low-middle SDI
Ireland	0.882181	High SDI

Israel	0.815944	High-middle SDI
Italy	0.843401	High SDI
Jamaica	0.678533	Middle SDI
Japan	0.865094	High SDI
Jordan	0.696845	Middle SDI
Kazakhstan	0.735474	High-middle SDI
Kenya	0.499472	Low-middle SDI
Kiribati	0.426768	Low SDI
Kuwait	0.785593	High-middle SDI
Kyrgyzstan	0.606647	Low-middle SDI
Laos	0.518789	Low-middle SDI
Latvia	0.825131	High SDI
Lebanon	0.729621	High-middle SDI
Lesotho	0.493357	Low-middle SDI
Liberia	0.328416	Low SDI
Libya	0.760934	High-middle SDI
Lithuania	0.840877	High SDI
Luxembourg	0.915748	High SDI
Macedonia	0.754364	High-middle SDI
Madagascar	0.330761	Low SDI
Malawi	0.349345	Low SDI
Malaysia	0.759249	High-middle SDI
Maldives	0.655287	Middle SDI
Mali	0.266901	Low SDI
Malta	0.835899	High SDI
Marshall Islands	0.550458	Low-middle SDI
Mauritania	0.470566	Low-middle SDI
Mauritius	0.720191	High-middle SDI
Mexico	0.628361	Middle SDI
Moldova	0.675573	Middle SDI
Mongolia	0.661854	Middle SDI
Montenegro	0.788189	High-middle SDI
Morocco	0.579231	Low-middle SDI
Mozambique	0.340471	Low SDI
Myanmar	0.555818	Low-middle SDI
Namibia	0.615792	Middle SDI
Nepal	0.428511	Low SDI
Netherlands	0.911855	High SDI
New Zealand	0.842274	High SDI
Nicaragua	0.529616	Low-middle SDI
Niger	0.190618	Low SDI
Nigeria	0.493394	Low-middle SDI
North Korea	0.53768	Low-middle SDI
Northern Mariana Islands	0.757817	High-middle SDI
Norway	0.910905	High SDI

Oman	0.743531	High-middle SDI
Pakistan	0.492158	Low-middle SDI
Palestine	0.541353	Low-middle SDI
Panama	0.677044	Middle SDI
Papua New Guinea	0.418998	Low SDI
Paraguay	0.61877	Middle SDI
Peru	0.635788	Middle SDI
Philippines	0.617174	Middle SDI
Poland	0.843773	High SDI
Portugal	0.777928	High-middle SDI
Puerto Rico	0.812984	High-middle SDI
Qatar	0.765716	High-middle SDI
Romania	0.784194	High-middle SDI
Russian Federation	0.791738	High-middle SDI
Rwanda	0.407441	Low SDI
Saint Lucia	0.652614	Middle SDI
Saint Vincent and the Grenadines	0.608304	Middle SDI
Samoa	0.576375	Low-middle SDI
Sao Tome and Principe	0.488258	Low-middle SDI
Saudi Arabia	0.779014	High-middle SDI
Senegal	0.373027	Low SDI
Serbia	0.751793	High-middle SDI
Seychelles	0.692334	Middle SDI
Sierra Leone	0.357159	Low SDI
Singapore	0.872215	High SDI
Slovakia	0.84169	High SDI
Slovenia	0.86028	High SDI
Solomon Islands	0.425019	Low SDI
Somalia	0.234807	Low SDI
South Africa	0.676543	Middle SDI
South Korea	0.871956	High SDI
South Sudan	0.274706	Low SDI
Spain	0.824617	High SDI
Sri Lanka	0.679706	Middle SDI
Sudan	0.477915	Low-middle SDI
Suriname	0.640993	Middle SDI
Swaziland	0.5777	Low-middle SDI
Sweden	0.88349	High SDI
Switzerland	0.888753	High SDI
Syria	0.611084	Middle SDI
Taiwan	0.864186	High SDI
Tajikistan	0.522612	Low-middle SDI
Tanzania	0.412207	Low SDI
Thailand	0.684277	Middle SDI

The Bahamas	0.755562	High-middle SDI
The Gambia	0.40476	Low SDI
Timor-Leste	0.504843	Low-middle SDI
Togo	0.413313	Low SDI
Tonga	0.624951	Middle SDI
Trinidad and Tobago	0.698405	Middle SDI
Tunisia	0.675429	Middle SDI
Turkey	0.729481	High-middle SDI
Turkmenistan	0.696419	Middle SDI
Uganda	0.387738	Low SDI
Ukraine	0.740062	High-middle SDI
United Arab Emirates	0.794722	High-middle SDI
United Kingdom	0.843093	High SDI
United States	0.866622	High SDI
Uruguay	0.706753	High-middle SDI
Uzbekistan	0.629547	Middle SDI
Vanuatu	0.475309	Low-middle SDI
Venezuela	0.655413	Middle SDI
Vietnam	0.606829	Middle SDI
Virgin Islands, U.S.	0.806569	High-middle SDI
Yemen	0.429504	Low SDI
Zambia	0.472213	Low-middle SDI
Zimbabwe	0.463196	Low-middle SDI

SDI: Socio-demographic Index.

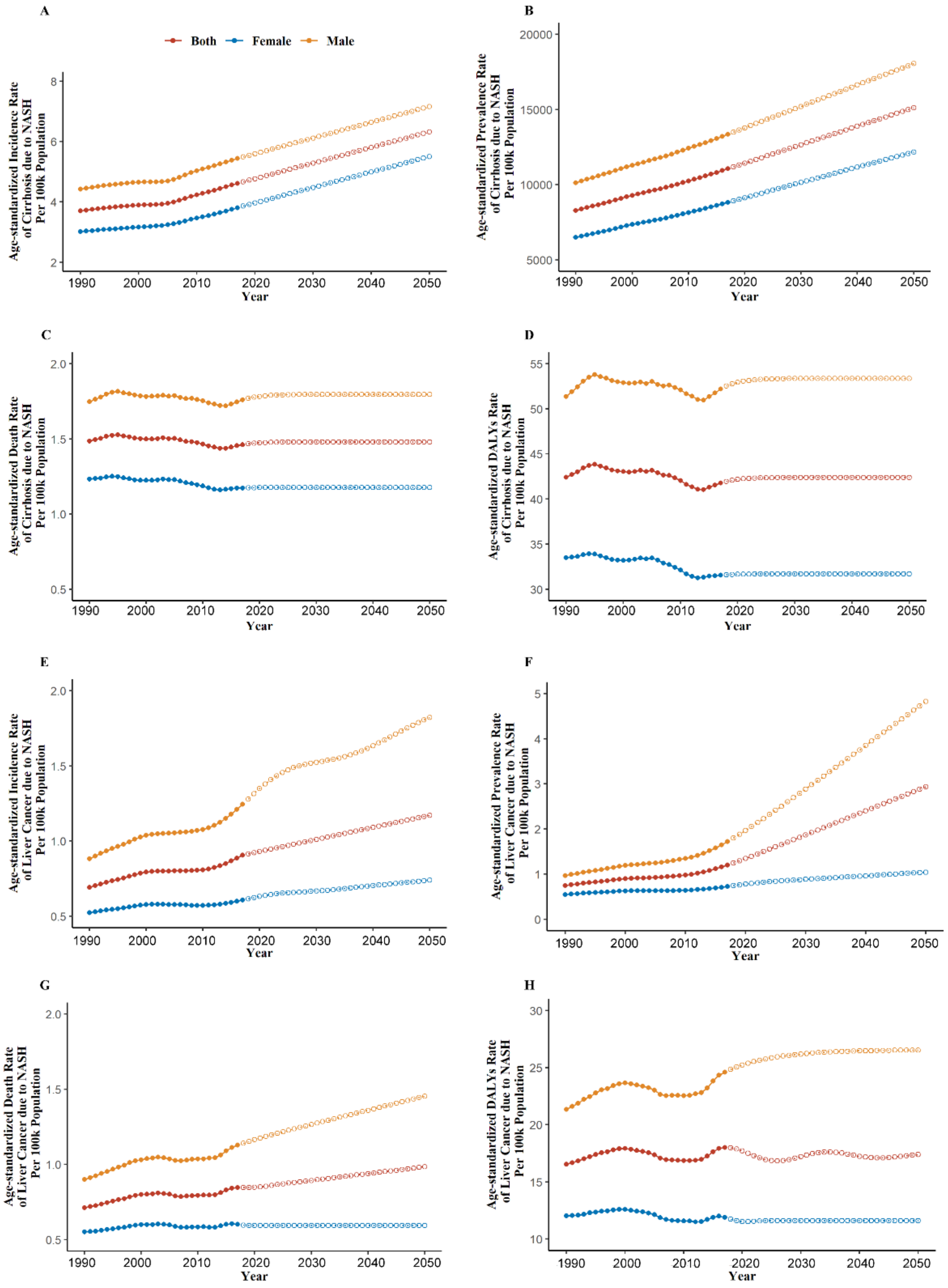

Figure S1 Global burden of cirrhosis and liver cancer due to NASH from 1990 to 2050. (A) Age-standardized incidence rate of cirrhosis due to NASH; (B) Age-standardized prevalence rate of cirrhosis due to NASH; (C) Age-standardized death rate of cirrhosis due to NASH; (D) Age-standardized DALYs rate of cirrhosis due to NASH; (E) Age-standardized incidence rate of liver cancer due to NASH; (F) Age-standardized prevalence rate of liver cancer due to NASH; (G) Age-standardized death rate of liver cancer due to NASH; (F) Age-standardized DALYs rate of liver cancer due to NASH. NASH: nonalcoholic steatohepatitis; DALYs: disability-adjusted life years. Dash line: forecasted NASH burden

from 2018 towards 2050 through ARIMA model.
